# Trichome-Related Mutants Provide a New Perspective on Multicellular Trichome Initiation and Development in Cucumber (*Cucumis sativus* L)

**DOI:** 10.3389/fpls.2016.01187

**Published:** 2016-08-10

**Authors:** Xingwang Liu, Ezra Bartholomew, Yanling Cai, Huazhong Ren

**Affiliations:** ^1^College of Horticulture, China Agricultural UniversityBeijing, China; ^2^Beijing Key Laboratory of Growth and Developmental Regulation for Protected Vegetable Crops, China Agricultural UniversityBeijing, China

**Keywords:** unicellular, *arabidopsis*, multicellular, cucumber, mutants, trichome-related genes, regulator pathway

## Abstract

Trichomes are specialized epidermal cells located in aerial parts of plants that function in plant defense against biotic and abiotic stresses. The simple unicellular trichomes of *Arabidopsis* serve as an excellent model to study the molecular mechanism of cell differentiation and pattern formation in plants. Loss-of-function mutations in *Arabidopsis thaliana* have suggested that the core genes *GL1* (which encodes a MYB transcription factor) and *TTG1* (which encodes a WD40 repeat-containing protein) are important for the initiation and spacing of leaf trichomes, while for normal trichome initiation, the genes *GL3*, and *EGL3* (which encode a bHLH protein) are needed. However, the positive regulatory genes involved in multicellular trichrome development in cucumber remain unclear. This review focuses on the phenotype of mutants (*csgl3, tril, tbh, mict*, and *csgl1*) with disturbed trichomes in cucumber and then infers which gene(s) play key roles in trichome initiation and development in those mutants. Evidence indicates that *MICT, TBH*, and *CsGL1* are allelic with alternative splicing. *CsGL3* and *TRIL* are allelic and override the effect of *TBH, MICT*, and *CsGL1* on the regulation of multicellular trichome development; and affect trichome initiation. *CsGL3, TRIL, MICT, TBH*, and *CsGL1* encode HD-Zip proteins with different subfamilies. Genetic and molecular analyses have revealed that *CsGL3, TRIL, MICT, TBH*, and *CsGL1* are responsible for the differentiation of epidermal cells and the development of trichomes. Based on current knowledge, a positive regulator pathway model for trichome development in cucumber was proposed and compared to a model in *Arabidopsis*. These data suggest that trichome development in cucumber may differ from that in Arabidopsis.

## Introduction

Plant trichomes are highly specialized epidermal protrusions that are located on the surfaces of leaves, stems, petioles, sepals, seed coats, and other aerial organs. Their diversity is almost as great as the number of species on which they are found. Morphologically, they can be unicellular or multicellular as well as secretory glandular or non-glandular (Hülskamp et al., 1998; Hülskamp, 2004; Tissier, 2012; Chen et al., 2014). In the model plant *Arabidopsis thaliana*, extensive studies have been performed on unicellular trichome development, especially on leaves (Hülskamp, 2004; Ishida et al., 2008; Pesch and Hülskamp, 2009). Classical molecular genetic approaches have identified several regulators that work in distinct developmental processes, such as trichome initiation/formation, endo-reduplication, and branch construction, and growth orientation (Schwab et al., 2000; Szymanski et al., 2000; Chen et al., 2014). The regulatory pathways of unicellular trichome development comprise both positive (mutants develop fewer trichomes) and negative (mutants develop more and/or clusters of trichomes) transcription factors (Ishida et al., 2008; Balkunde et al., 2010; Grebe, 2012). The crucial positive transcription factors belong to three protein classes: one WD40-repeat protein TRANSPARENT TESTA GLABRA1 (TTG1) (Galway et al., 1994; Walker et al., 1999); four basic helix-loop-helix (bHLH) proteins: GLABRA3 (GL3)m ENHANCER OF GLABRA3 (EGL3) (Payne et al., 2000; Zhang et al., 2003), TRANSPARENT TESTA (TT8) (Zhang et al., 2003), and MYC-1 (Zhao et al., 2012); and three R2-R3 type-MYB transcription factors: GLABRA1 (GL1), MYB23 and MYB5 (Oppenheimer et al., 1991; Li et al., 2009; Tominaga-Wada et al., 2012). They bind together to form an MYB-bHLH-WD40 (MBW) trimeric complex that activates the downstream gene GL2 (*GLABRA2*), which initiates trichome differentiation (Pesch and Hülskamp, 2009). Moreover, several small, single-repeat MYB-negative regulatory proteins, such as TRIPTYCHON (TRY), CAPRICE (CPC), ENHANCER OF TRYAND CPC1 (ETC1), ECT2, ETC3, CAPRICE-LIKE MYB3, and TRICHOMELESS1, and TRICHOMELESS2 (TCL1, and TCL2, respectively), have been shown to act in a non-cell-autonomous manner (Wada et al., 1997, 2002; Esch et al., 2004; Kirik et al, 2004; Pesch and Hülskamp, 2009; Wester et al., 2009; Edgar et al., 2014; Hauser, 2014; Wang and Chen, 2014). They compete with the R2R3 MYB protein GL1 and bind to bHLH proteins, including GL3/EGL3, to suppress trichome initiation in adjacent cells (Wang et al., 2014).

Cucumber (*Cucumis sativus* L.), as one of the most important vegetable crops, is also covered with trichomes ranging from the stems and leaves to the flowers, branches, fruits, and tendrils. During early fruit development, deep ridges along the length of the fruit cover the fruit surface, and densely spaced fruit trichomes are randomly scattered relative to the ridges (Liu et al., 2011; Chen et al., 2014). Trichomes on cucumber fruit are called spines. In cucumber, the fruit spine combines with tubercles to form the warty fruit trait, which is a very important fruit quality trait (Chen et al., 2014; Li et al., 2015). Compared to warty fruit, smooth fruit, with no spines, or tubercles, is very important for the breeding of freshly eaten cucumber types, as they are easier to clean, package, transport and store (Zhang et al., 2010; Yang et al., 2014). Moreover, smooth fruit is becoming increasingly popular due to its attractive and distinctly shiny appearance (Li et al., 2015; Pan et al., 2015; Cui et al., 2016). Despite the importance of fruit spines in cucumber breeding for external quality, there are limited reports of the regulation of fruit spine development and few detailed characterizations of cucumber genes with disturbed trichome development. To understand trichome development in cucumber, we must determine the crucial regulators for its initiation and formation based on cucumber trichome-related mutants.

We begin this review by focusing on cucumber trichome-related mutants and the role that they play in trichome formation in plants with multicellular trichomes. Several mutants, such as *trichome-less* (*tril*) (Wang et al., 2016), *glabrous 3* (*csgl3*) (Pan et al., 2015; Cui et al., 2016), *tiny branched hair* (*tbh*) (Chen et al., 2014), *micro-trichome* (*mict*) (Zhao et al., 2015a), and *glabrous 1* (*csgl1*) (Li et al., 2015), inhibit trichome development via different mechanisms, but all of these mutants cause a reduction in one type of spine. The possible mechanisms involved in modulating trichome development in cucumber mutants are summarized herein.

## Mutants with disturbed trichome in cucumber

**Figure 1:**
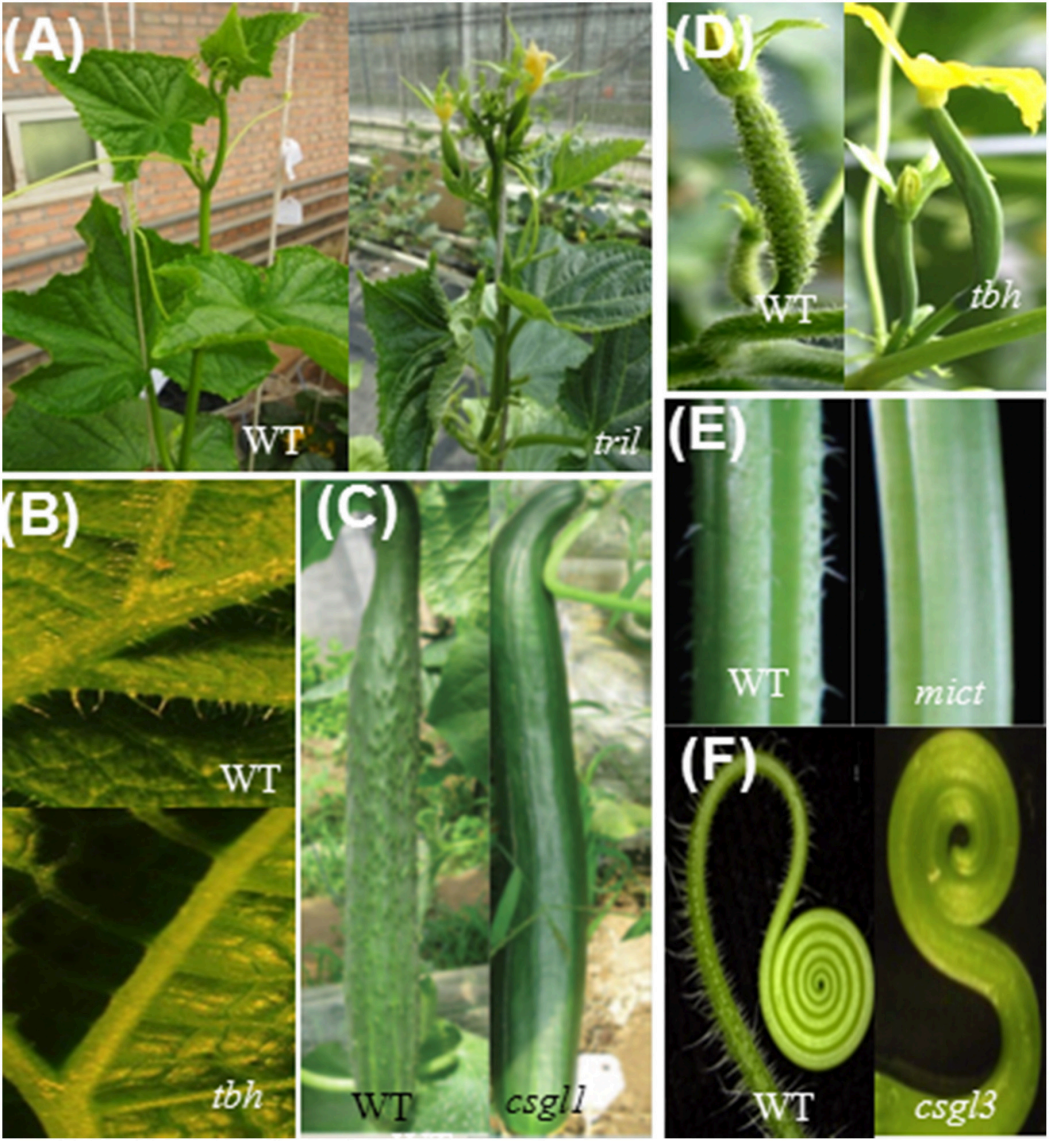
**Phenotypic characterization of mutants and the wild type (WT)**. **(A)** WT and *tril* plants; **(B)** Leaves; **(C)** Fruits; **(D)**. Fruit 0 days after anthesis; **(E)** Stems; **(F)** Tendril. Picture **(A)** is from Wang et al. (2016); Pictures **(B,D)** are from Chen et al. (2014); and Pictures **(C,E)** are from Li et al. (2015); Picture **(F)** cited form Pan et al. (2015).

**Figure 2:**
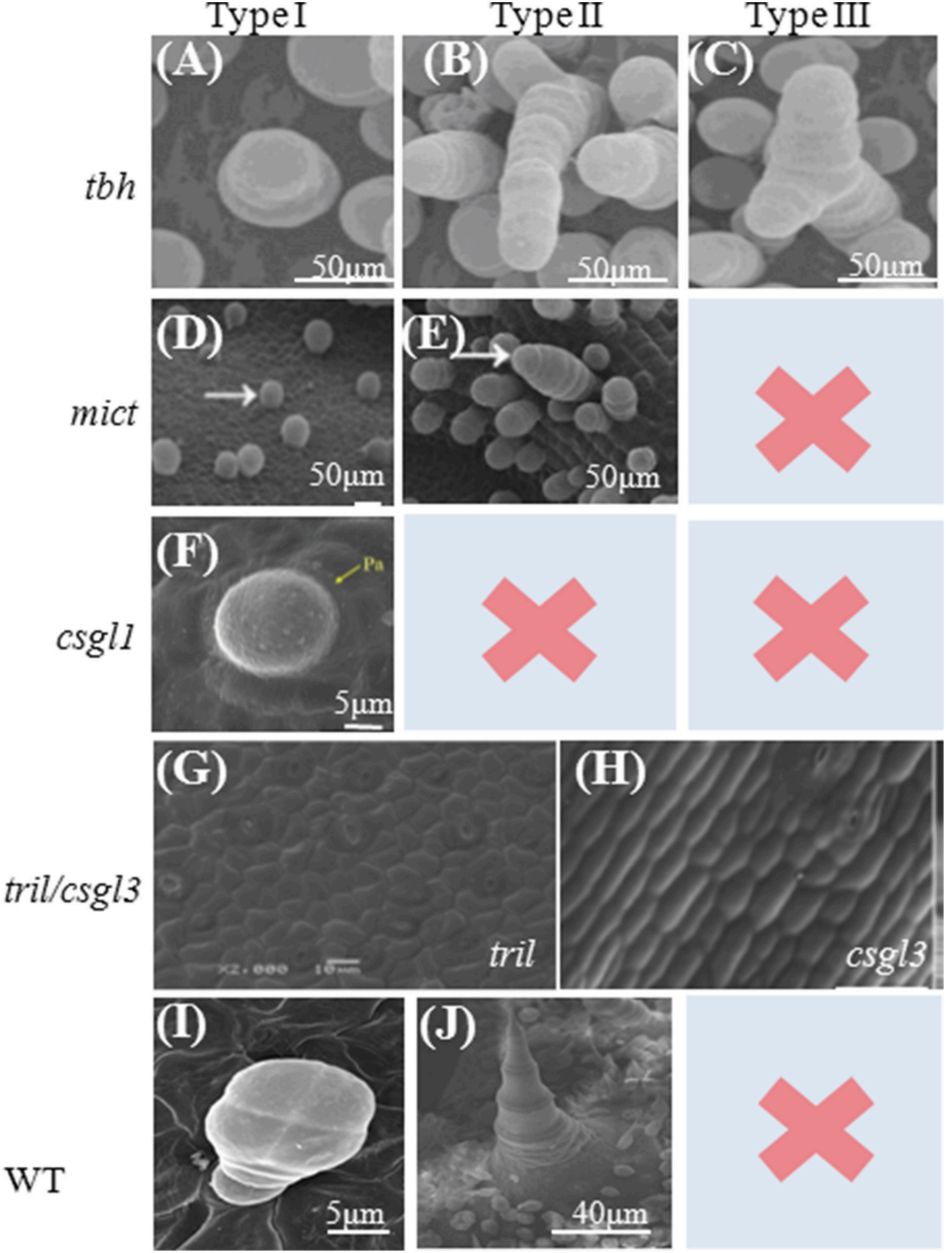
**Scanning Electron Microscopy images of trichomes of cucumber mutants**. **(A–C)** SEM images of fruit spines **(A,B)** and leaf trichomes **(C)** in *tbh*, three morphologies from left to right; **(D–E)**. Micro-trichomes on a *mict* leaf. Arrows indicate the two morphologies: type I (right) and type II (left), respectively; **(F)**. Trichomes on the epidermis of leaves from *csgl1*; **(G,H)**. The *tril/csgl3* mutant has a completely glabrous phenotype, without trichome material in fruit epidermis. Trichome on the Fruit from the Wild type as control **(I,J)**. Pictures **(A–C)** are from (Chen et al., 2014); Pictures **(D,E,H)** are from (Zhao et al., 2015a); Picture **(F)** is from (Li et al., 2015), and Picture **(G)** is from (Wang et al., 2016).

Five trichome-related mutants have been reported in cucumber (Chen et al., 2014; Li et al., 2015; Pan et al., 2015; Zhao et al., 2015a; Cui et al., 2016; Wang et al., 2016). Wild-type cucumber fruits have two types of trichomes, both of which are multicellular (Figures 1, 2) (Chen et al., 2014). Type I trichomes are small, glandular trichomes, with a 3- to 5-cell base topped with a 4- to 8-cell head. Type II trichomes, which predominate, are much larger, non-glandular trichomes that are composed of a base and stalk (Figure 2). Compared to wild-type cucumber plants, all mutants appeared to be glabrous with no noticeable trichomes on the leaves, stems, tendrils, floral organs, or fruits (Table 1, Figures 1, 2). Each mutant has a trichome type except for the mutants *tril* and *csgl3* (Figure 2). Trichomes have been reclassified into three morphologies according to their shape on leaves and fruit surfaces. Type I trichomes, which are found in the *tbh, mict*, and *csgl1* mutants, have a small papillar-shaped head (Figures 2A,D,F). Type II trichomes, which exist in the *tbh* and *mict* mutants, consist of one to five rounded cells without the pyramid-shaped head or pie-shaped base (Figures 2B,E). However, type III trichomes occur only in *tbh* mutants; this type of trichome was not previously described. Here, we classified these trichomes into the new type III according to the branching at the top of the trichome (Figure 2C). Trichome phenotypes from *tbh, mict* and *csgl1* indicated that these three mutants are all involved in trichome development and not in trichome initiation. Interestingly, the *tril* and *csgl3* mutants showed a completely glabrous phenotype on the shoot and fruit epidermis (Figures 2G,H), suggesting that *TRIL* and *CsGL3* may be upstream positive regulators of *TBH, MICT*, and *CsGL1* for the regulation of multicellular trichome development and may affect epidermal cell initiation.

**Table 1:** **Previous morphological studies of trichome in *tbh, csgl1, mict, tril and csgl3***.

**Mutant name**	**Origin**	**Types of trichome described**	**References**
*tbh*	Northern China	Type I: tiny 3- to 5-cell base topped with a 4- to 8-cell head Type II: non-glandular and branchless	Chen et al., 2014
*mict*	Northern China	Type I: only a small papillar-shaped head Type II: 1-5 cells without the pyramid shaped head	Zhao et al., 2015a,b
*csgl1*	Northern China	No trichomes on leave except glandular trichomes	Li et al., 2015
*tril*	European greenhouse	No any types trichome	Wang et al., 2016
*csgl3*	European greenhouse	No any types trichome	Pan et al., 2015

Compared to the multicellular trichomes found on aerial organs, such as leaves and fruits, the trichomes found on underground organs, such as root hairs, are characterized as single-celled, unbranched, elongated, and soft-structured with small tumors attached. Based on the published results, there was no difference between the wild-type and the *tbh, mict*, and *csgl1* mutants (Chen et al., 2014; Li et al., 2015; Zhao et al., 2015a), indicating that the *TBH, MICT*, and *CsGL1* mutants may not be involved in root hair formation. In contrast, the root length and number of branches of the *tril* mutant increased, suggesting that root hair formation in cucumber might be regulated by *TRIL* (Wang et al., 2016).

Other remarkable differences (except for the common phenotype) may exist among each mutant, such as dwarfism, branching, leaf curvature, and petal opening rates. In *mict* and *tbh*, rounded-head trichomes were found on the hypocotyls, but none were found in *csgl1*, suggesting trichome distribution is specific (Chen et al., 2014; Li et al., 2015; Zhao et al., 2015a).

## *TBH, MICT*, and *CsGL1* are the allelic with alternative splicing

**Figure 3:**
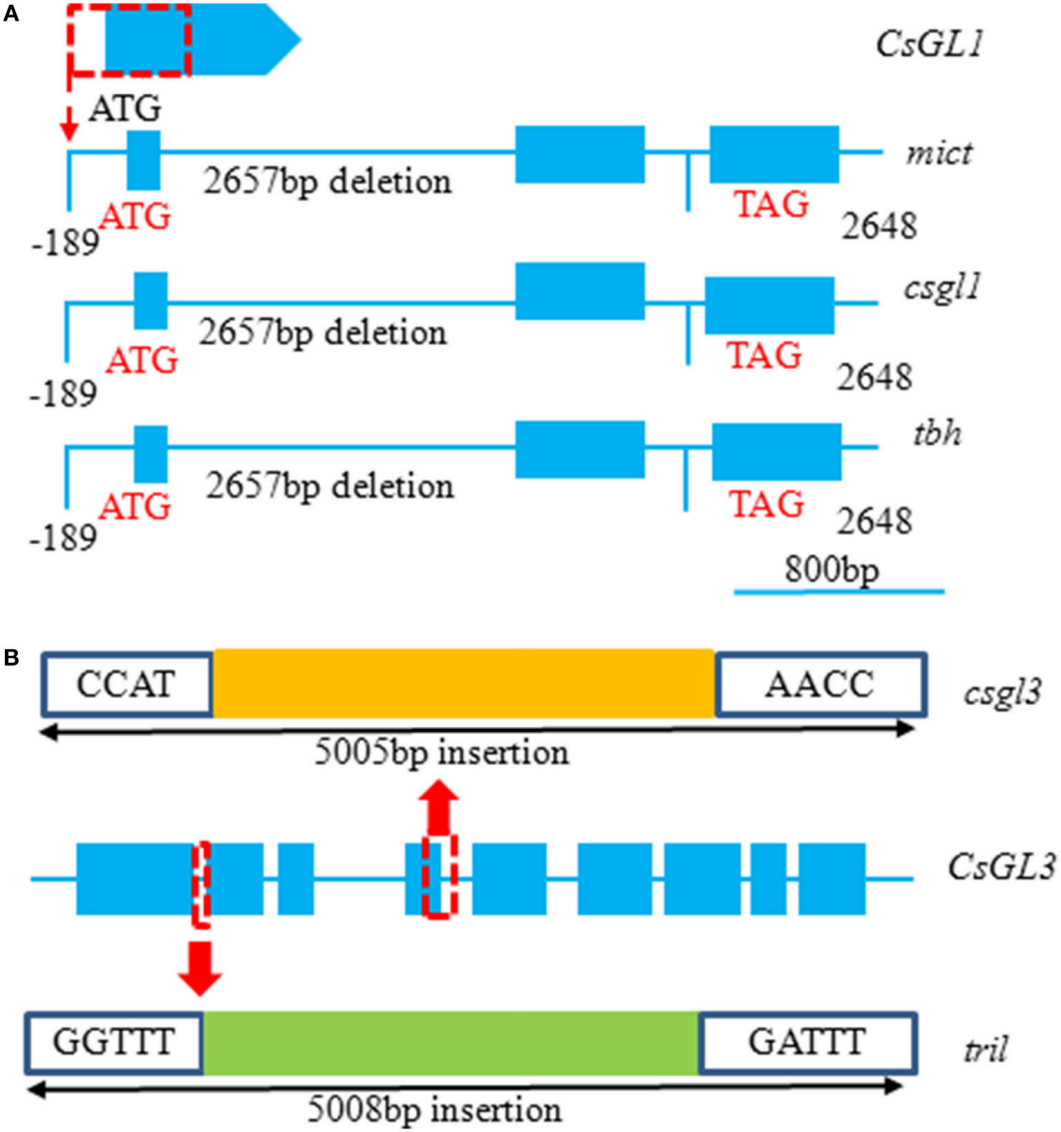
**Gene structure of *CsGL1* and *CsGL3* in the WT and mutants**. **(A)** A 2649 bp deletion of the start codon in *CsGL1* in the *mict, csgl1 and tbh* mutants compared to the WT. Boxes represent exons, and lines represent introns and intergenic regions. ATG and TAG indicate start and stop codons, respectively, and numbers represent nucleotide deletion length in mutants. *Mict* is from Zhao et al. (2015a); *csgl1* is from Li et al. (2015). **(B)** A 5005 bp insertion at the 4th exon in *csgl3* and a 5008 bp insertion after the first exon in the *tril* mutant. The red box indicates insertion sites, and yellow and green boxes represent insertion length. Different insertions in this gene showed the same phenotype in cucumber. *tril* is from Wang et al. (2016); *csgl3* is from Pan et al. (2015).

To decipher the molecular defects in the *mict, csgl1*, and *tbh* mutants, a map-based cloning approach was undertaken by three independent groups to isolate these genes. The results showed that *TBH, MICT*, and *CsGL1* are allelic and that there was a 2649 bp fragment deletion from -189 to 2460 bp of the start codon in *Csa3M748220* in the *mict, csgl1*, and *tbh* mutants (Li et al., 2015; Zhao et al., 2015a) (Figure 3A). According to the gene ID that provided in the references, we extracted their proteins from the cucumber genome database (http://www.icugi.org/cgi-bin/ICuGI/genome/search.cgi) and analyzed their protein domains online (https://blast.ncbi.nlm.nih.gov/Blast.cgi). These genes were predicted to encode a class I homeodomain-leucine zipper (HD-Zip) protein consisting of different amino acid residues. For example, CsGL1 and TBH contain 240 amino acids with the conserved HD (65AA-120AA) and Zip domains (121AA-164AA), but the MICT protein consists of 242 amino acid residues with a HALZ motif. Notably, there were two isoforms of this gene in the cucumber genome database (Csa3M748820.1 and Csa3M748820.2); therefore, we infer that the different protein lengths encoded by the same gene suggest that *Csa3M748220* may exist by alternative splicing in cucumber. Subcellular localization showed that the *Csa3M748220* coding sequence was fused to GFP (35S) in the nuclei of tobacco and onion epidermal cells (Zhao et al., 2015a,b). Moreover, a transcriptional activation activity assay in yeast found that *Csa3M748220* had weak activity as a transcriptional activator (Zhao et al., 2015a). Therefore, based on the above results, *Csa3M748220* has the typical features of a transcription factor. HD-Zip proteins are unique to the plant kingdom and can be classified into four groups, I-IV, based on their distinctive traits of DNA-binding specificities, gene structures, and common motifs (Abe et al., 2003; Hülskamp et al., 2005). HD-Zip I genes have been demonstrated to be involved largely in biological processes, such as abiotic stress responses, meristem regulation, and trichome development (Hanson et al., 2001; Himmelbach et al., 2002; Hjellström et al., 2003; Saddicl et al., 2006; Zhao et al., 2015a,b).

## *TRIL, CsGL3* are the allelic which override the effect of *TBH/MICT/CsGL1* on the regulation of multicellular trichome initiation and development

Thereare few reports on the regulatory genes that control multicellular trichome development in cucumber. This presents a good opportunity to use these mutants to analyze their regulatory mechanisms in trichomes of cucumber. As mentioned above, *tril* and *csgl3* show a completely different glabrous morphology from that of the other three trichome-development-related mutants, and only the epidermal cells, including stomata and encircling guard cells, were visible (Figure 2G). This indicates that *TRIL* and *CsGL3* function in trichome cell fate determination. A transcriptome profiling analysis among wild-type, *tril, tbh, mict*, and *csgl1* revealed that *TBH, MICT*, and *CsGL1* were not expressed in the *tril* mutant (baseMean 0) but were highly expressed in the wild-type (baseMean 518.25) (Zhao et al., 2015a). *TRIL* was mapped to *Cas6M514870*, a member of the class IV HD-Zip family that shares 66.7% identity with *PROTODERMAL FACTOR2* (*PDF2, At4g04890.1*), a shoot epidermal cell differentiation-related gene, and 35% identity with GL2 (*At1g79840.2*), a gene that initiates trichome differentiation in *Arabidopsis*. In the *tril* mutant, there is a 5008 bp insertion fragment after the first exon (Figure 3B). *CsGL3* was also mapped to *Csa6M514870* by Pan and Cui (Pan et al., 2015; Cui et al., 2016). In Pan's study, the loss of function of the *CsGL3* was due to the insertion of a 5-kb-long terminal repeat (LTR) retrotransposon in the 4th exon of *CsGL3* (Figure 3B). The insertion location is different from that of *tril*. Cui used three markers (InDel-19, dCAPs-2, and dCAPs-11) designed from the sequence of *Csa6M514870*, which co-segregated with the trait. In addition, *Csa6M514870* was found to harbor 3 single-base substitutions: T → C (611 bp), G → A (820 bp), and G → A (865 bp) at the fourth exon, resulting in a change in the amino acid sequence. We still do not know how the *CsGL3* changed in its corresponding mutant *csgl3*.

In the *tbh, mict*, and *csgl1* mutants, *TRIL/CsGL3* was over-expressed at different levels due to the different stages at which the samples were collected (Chen et al., 2014; Li et al., 2015; Zhao et al., 2015a). Moreover, the trichome phenotype analyzed in the F2 population between the *tril* and *mict* mutants suggests that the *TRIL* gene has a significant influence on trichome initiation and in determining the fate of epidermal cells, whereas the *MICT*/*TBH/CsGL1* gene only influences trichome development at the shoot. Genetically, based on the double-mutant phenotype in *csgl3csgl1*, the *TRIL/GsGL3* gene is assumed to act upstream of the *MICT*/*TBH/CsGL1* gene and control the expression of *MICT*/*TBH/ CsGL1* (Zhao et al., 2015a; Wang et al., 2016).

## Models for trichome patterning in cucumber may differ from models in *Arabidopsis*

**Figure 4:**
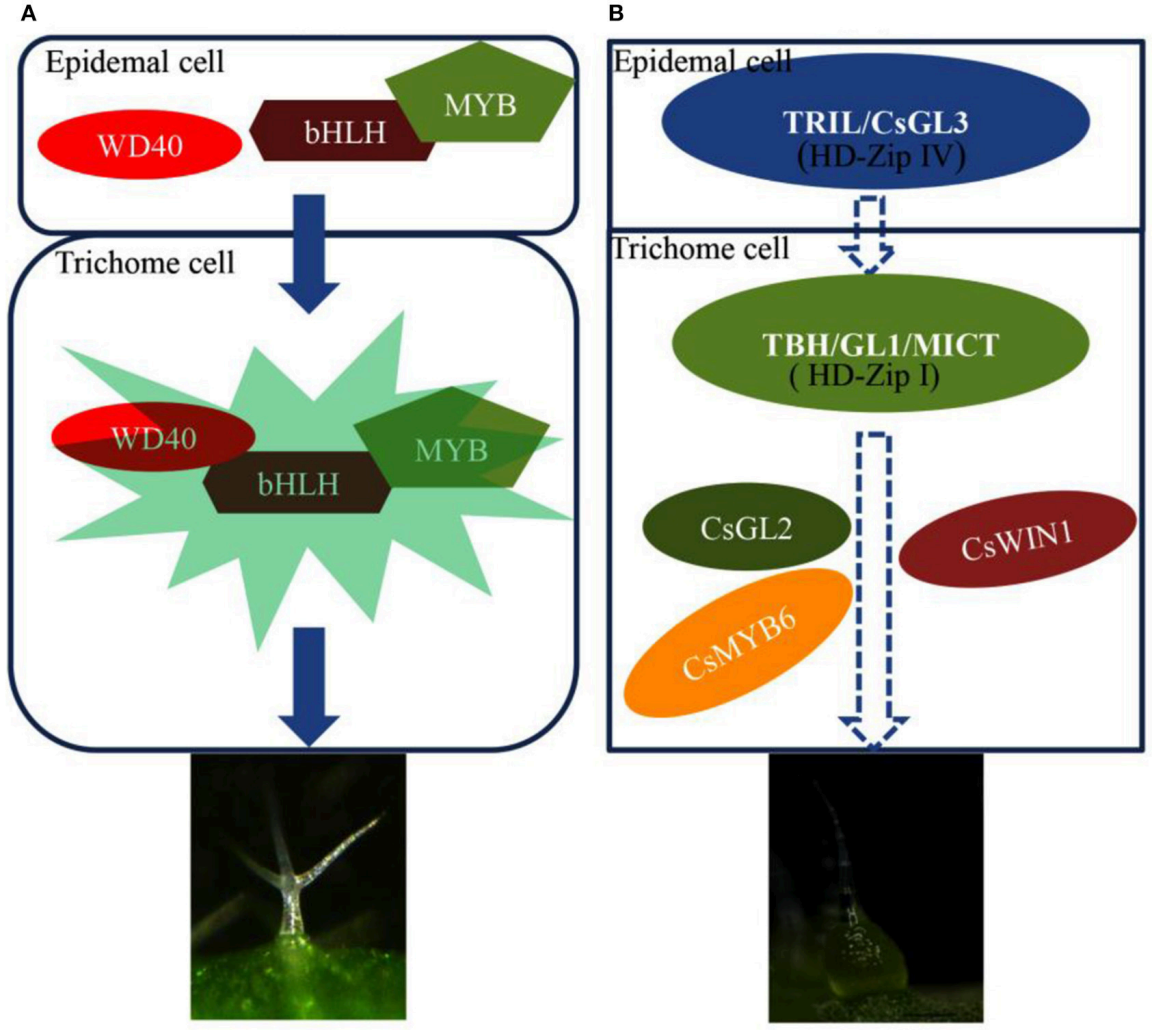
**Proposed model to explain trichome patterning in the positive regulator pathway in cucumber and in *Arabidopsis*. (A)** *TRIL/CsGL3* encodes the HD-Zip IV protein and may directly interact with the HD-Zip I protein (MICT/TBH/CsGL1) to regulate trichome development in cucumber. *CsMYB6, CsWIN1*, and *CsGL2* may be located downstream of *MICT/TBH/CsGL1* and positively participate in trichome development.**(B)** In unicellular trichomes, WD40, Bhlh, and MYB form a complex to positively regulate trichome development. The core complex in MBW **(A)** may differ from the HD-Zip complex **(B)**. The bold arrows in the boxes indicate developmental progression.

In the model plant *Arabidopsis*, the activator-inhibitor model has guided intuitive modeling and experimental design for a long time because it offers a reasonable explanation for the apparently paradoxical situation in that trichome-promoting and trichome-inhibiting genes are both expressed strongly in trichomes (Pesch and Hülskamp, 2009). Here, we focus on a positive regulatory model for trichome development between *Arabidopsis* and cucumber. The main reason is that all of the mutants in cucumber reveal fewer or no trichomes visually, suggesting that they encode a positive regulator of trichome development. Solid evidence of the genetic basis of trichome initiation has identified genes that (a) control the entry into the trichome pathway and (b) control the spacing of initiation events. Loss-of-function mutations in *Arabidopsis thaliana* have suggested that the genes *GL1* (which encodes an MYB transcription factor) and *TTG1* (which encodes a WD40 repeat-containing protein) are important for the initiation and spacing of leaf trichomes (Galway et al., 1994; Walker et al., 1999), while for normal trichome initiation, the genes GL3, and EGL3 (Payne et al., 2000; Zhang et al., 2003), which encode helix-loop-helix (bHLH) proteins, are needed (Figure 4A).

In cucumber, the *TRIL/CsGL3* gene encodes a HD-Zip IV protein. The loss-of-function mutant showed that *tril*/*csgl3* has a trichome-less phenotype, indicating that this gene is a positive regulator of trichome development. Its homologous genes PDF2 and *ARABIDOPSIS THALIANA MERISTEM LAYER1* (*ATML1*) in *Arabidopsis* also encode HD-Zip IV protein family members (Nakamura et al., 2006). The *pdf2atml1* double mutant displays striking defects in shoot epidermal cells (Abe et al., 2003). In tomato, another typical multicellular trichome plant, *Wo* RNAi transgenic tomato plants showed similar shoot and root epidermal cell formation as that of the *tril* mutant. *Wo* shares an amino acid sequence identical to that of *TRIL/CsGL3* and belongs to the same HD-Zip IV protein family. These results indicate that the role of *TRIL/CsGL3* in the initiation and control of multicellular trichrome pathways. Based on previous results, a proposed model explaining trichome patterning in the positive regulator pathway in cucumber was built (Figure 4B). In this model, HD-Zip transcription factors may bind to other types of transcription factors to generate a specific complex to control cucumber multicellular trichome formation.

Based on transcriptional data from all of the mutants, several candidate genes should be focused on extensively (Chen et al., 2014; Zhao et al., 2015a,b). For example, the *CsMYB6, CsWIN1*, and *CsGL2* genes were down-regulated not only in *tril/csgl3* but also *in tbh, mict*, and *csgl*, indicating that those genes were involved in multicellular trichome development in cucumber.

## Discussions

In the past decade, substantial progress has been made in delineating the genes that control trichome development in cucumber. Several groups provide evidence to suggest that a number of transcriptional activators, such as HD-Zip I and HD-Zip IV, play a role in fine tuning the spatial and temporal distribution of trichomes. Researchers have tried to demonstrate the possible mechanisms of these transcriptional activators.

However, much remains unknown and needs to be elucidated in future research. The functions of the HD-Zip IV and HD-Zip I genes require further investigation through genetic transformation in cucumber plants. Our current knowledge about the gene regulatory networks is largely limited to the unicellular trichomes in the model plant *Arabidopsis*. However, little is known about the regulatory network that controls the development of multicellular trichomes in cucumber. Evidence indicates that the common regulatory mechanisms of unicellular trichomes in *Arabidopsis* or of multicellular trichomes in cotton involve plant-specific genes that function distinctively. An analysis of the differential expression data generated by RNA-seq can offer new information for identifying putative key multicellular development transcription factors in cucumber. This is why we focused on the critical transcription factor genes. A new set of more than 42 transcription factor genes, including Homeodomain, MCMI-AGAMOUS-DEFICIENS-SRF4 (MADS), and WRKY domains, has been identified in a transcriptome analysis of all cucumber trichome-related mutants (Zhao et al., 2015a). These transcription factor genes are unique to plants and are involved in a range of activities; many are associated with multicellular trichrome development and other species-specific development processes. The study of the interaction among those transcription factors is an emerging area of research because these factors probably share many biological functions for trichome development. All of this information warrants further investigation.

The aim of this review is to provide readers with a summary of the progress of cucumber trichome development and to encourage plant scientists to further investigate the mechanism of trichrome initiation and development and their regulatory network. We have summarized the mutants related to fruit trichomes, the key genes that control trichome initiation and development, gene relationships and a possible model for fruit trichome positive regulatory mechanisms that is different from *Arabidopsis* in core transcriptional factor numbers. All of this may help us to better understand the advances in the study of cucumber trichomes. At the same time, more investigations must be conducted.

Because cucumber is a horticultural crop of worldwide importance and fruit spines directly affect its commercial quality, an extensive characterization of cucumber trichomes will not only help us to understand the underlying molecular mechanisms involved in multicellular trichome development but will also pave the way for creating new cucumber varieties with desired trichome growth and density. Moreover, cucumber trichomes may serve as a model system for studying the development of multicellular trichomes.

## Author contributions

HR and XL organized the review, wrote the first draft and generated Figures 1–4. EB and YC contributed to a second draft. All of the authors revised the manuscript multiple times. HR and XL performed the final revision of the manuscript, which was read and approved by all authors.

### Conflict of interest statement

The authors declare that the research was conducted in the absence of any commercial or financial relationships that could be construed as a potential conflict of interest.

## Abbreviations

TRIL: trichome-less
TBH: tiny branched hair
MICT: micro-trichome
TTG1: transparent testa glabra1
bHLH: basic helix-loop-helix
GL3: glabra3
EGL3: enhancer of glabra3
MBW: MYB-bHLH-WD40
TRY: triptychon
CPC: caprice
ETC1: enhancer of tryand caprice1
HD-Zip: homeodomain-leucine zipper
PDF2: protodermal factor2
ATML1: *Arabidopsis thaliana* meristem layer1
MADS: MCMI-AGAMOUS-DEFICIENS-SRF4.
